# On the morphology, biometry and biogeography of *Lamtopyxis callistoma* (Amoebozoa: Arcellinida)

**DOI:** 10.3897/BDJ.3.e4297

**Published:** 2015-01-13

**Authors:** Milcho Todorov

**Affiliations:** † Institute of Biodiversity and Ecosystem Research, Bulgarian Academy of Sciences, 2 Gagarin Str., 1113 Sofia, Bulgaria

**Keywords:** Testate amoebae, Amoebozoa, Arcellinida, *Lamtopyxis callistoma*, ultra-structure, biometry, biogeography.

## Abstract

The ultra-structure of the shell and the morphometric variability of soil inhabiting testate amoeba *Lamtopyxis callistoma* from Madagascar were studied by using light- and scanning electron microscopy. The biometrical characteristic of the species was made on the basis of 75 specimens measured. In addition to the diameter of the shell, six other shell characters were described biometrically for the first time. The analysis of the variation coefficients shows that the studied population of *L. callistoma* is comparatively homogeneous and almost all measured characters are weakly to moderate variable (CV less than 10%). Scanning electron microscopy (SEM) studies on the shell ultra-morphology show that it has a smooth apertural surface with a thick layer of porous and fibrous organic cement and a rough dorsal surface composed of bigger and angular pieces of quartz. The shell wall has a thickness of about 5-6 µm and is composed of three layers. Unlike the previously accepted opinion that species is characterized by the presence of four teeth, this study shows that population of *L. callistoma* from Madagascar is comprised of both, specimens with four teeth and specimens with three teeth, in ratio of about 60% to 40%. Taking into account the restricted geographical distribution, large sizes and characteristic apertural morphology of *L. callistoma* it is assumed that this species, like some bryophilic ‘Nebelas’ with circumaustral distribution (e.g. *Apodera vas*, *Alocodera cockayni*, *Certesella certesi*, *Certesella martiali*, etc.), can be used as an example that in free-living microbial eukaryotes ‘not everything is everywhere’.

## Introduction

The genus *Lamtopyxis* is one of the testacean genera which include exclusively soil inhabiting species. It was described by Bonnet (1974) and so far only 5 species are known (*L. callistoma* Bonnet, 1974, *L. cassagnaui* Bonnet, 1977, *L. travei* Bonnet, 1977, *L. trifoliata* Bonnet, 1979 and *L. sarocchii* Coûteaux et Chardez, 1981). A typical characteristic for the species of this genus is specific apertural morphology with presence of 3-5 apertural teeth. Another feature is that they have been found only in soils of tropical forests from countries situated south of the Tropic of Cancer: Nepal (Bonnet 1977c, Bonnet 1981b), Mexico (Bonnet 1977a), Brazil and Paraguay (Bonnet 1979b), Philippines (Bonnet 1980b), French Guiana (Coûteaux and Chardez 1981), Thailand (Bonnet 1981a, Bonnet 1987, Golemansky and Todorov 2000), Indonesia (Bonnet 1985, Bonnet 1992), Ecuador (Krashevska et al. 2007, Krashevska et al. 2010). This is the reason that they are considered a rare species of the Gondwanan-tropical group, with restricted geographical distribution. Because of their rarity and the fact that they have been described using mainly light microscopy, data about their ultramorphology and biometry are incomplete in the literature. *L. callistoma* is a type species of the genus which was first described by Bonnet (1974) from gallery forest from Ivory Coast (the Savanna Lamto). Until now it has been recorded from only four tropical countries: Ivory Coast (Bonnet 1974, Bonnet 1976), Papua New Guinea (Bonnet 1980a), Philippines (Bonnet 1980b) and Indonesia (Bonnet 1992) and its ultramorphology and biometry is still poorly studied. During our investigation on the soil testate amoebae from Madagascar we observed an abundant material of *L. callistoma*. This provided us the opportunity to make more detailed studies on the shell ultramorphology and morphometric variability of this species. The aim of the present study was to describe morphologically and biometrically *L. callistoma* and to assess the biogeographical importance of this rare species of the Gondwanan-tropical group of soil testate amoebae.

## Materials and methods

The material for the present study (an aggregate sample of about 500 g) was collected in October 2013 from the organic horizon (litter, twigs and woody material) of rainforests at the Maromizaha Protected Area (Madagascar). This Protected Area is located in Central Madagascar, east of the capital Antananarivo and south of the village Anevoka, on the eastern slopes of the Central Highlands (18°57'S, 48°27'E, 950 m a.s.l.). At the laboratory, the material was dried for one day in a thermostat at 60˚C. Then was soaked and mixed in chlorinated tap water for about ten minutes, after which was filtered through a sieve with 500 μm mesh to remove large organic and mineral particles. The resulting filtrate was allowed to precipitate for two hours, the sediment was removed and the shells that floated on the surface were collected for examination. The study was carried out 12 hours after the flotation, when the gas bubbles inside the shells completely disappeared and the structure of the shells was well visible. All filtrate was examined in a petri dish at 100X magnification with Stereomicroscope “STEMI” Citoval-2 (Carl Zeiss Jena). A total of 81 individuals of *L. callistoma* were determined and isolated. The basic morphometric characters of 75 of them were measured by an optical microscope “Amplival” (Zeiss-Jena, Germany) at 400X magnification. The isolated specimens were transferred in a drop of glycerol in order to maintain them in a given position during the measurements.

Remaining six specimens were studied by scanning electron microscope (SEM). They were extracted using a glass micropipette, washed several times in distilled water, and then individual shells were positioned with a single-hair brush onto a small drop of Araldite on a previously cleaned standard aluminium stub and air-dried. The shells were coated evenly with gold in a vacuum coating unit. The photomicrographs were obtained using a JEOL JSM-5510, operating at 10 kV.

The biometric description was made according to Schönborn et al. (1983). Frequency distribution analysis was carried out in order to describe variation of characters. Statistical analysis was performed using the computer program STATISTICA, version 7.0 (StatSoft 1999).

## Taxon treatments

### Lamtopyxis callistoma

Bonnet, 1974

#### Materials

- **Type status:** Other material. **Occurrence:** recordedBy: Milcho T. Todorov; individualCount: 75; **Location:** country: Madagascar; stateProvince: Maromizaha Protected Area; verbatimLocality: Central Madagascar, east of the capital Antananarivo and south of the village Anevoka, on the eastern slopes of the Central Highlands; verbatimElevation: 950 m; verbatimLatitude: 18°57'S; verbatimLongitude: 48°27'E; **Record Level:** collectionID: col:ta;prep.24,25,26/2014; institutionCode: IBER; collectionCode: Testate amoebae

#### Description

The shell is yellowish or light brown, circular in oral and dorsal views, and hemispherical in lateral view (Figs 1, 2). The apertural surface is smooth, composed of small to medium flattish mineral particles, bound together by a thick layer of porous and fibrous organic cement, which extends over the teeth (Figs 2a, b, c, 3a). The dorsal surface is rough and composed of bigger and angular pieces of quartz, bound together by considerably less amount of organic cement (Figs 2d, e, f, 3c). The shell wall has a thickness of about 5-6 µm and is composed of three layers: an outer layer of organic cement (300-500 nm) with a smooth outer lining, an intermediate layer of porous and fibrous organic cement (2-4 µm) with incorporated mineral particles and an inner layer of organic cement (1-1.5 µm) with a rough inner lining (Fig. 3e, f). Small single pores with a varying diameter of about 50-350 nm are often seen in the cement, in both on the apertural and dorsal shell surface (Fig. 3b). The aperture is central, deeply invaginated (about 40% of the shell depth), with pronounced apertural tube and two openings - external and internal (Figs 1c, d, 3d, e). The external opening is more often four-, less often three-lobed, bordered by 3-4 large and smooth teeth (Figs 1a, b, 2a, b, c). The internal opening is at the bottom of the apertural tube. It varies in shape from oval to almost circular and is surrounded by a small collar (Figs 1a, 3a). The apertural tube is reinforced at its base by a characteristic dark-brown coloured organic frame (visible by LM, but not by SEM) (Fig. 1a). The apertural tube is rough, composed of a mixture of medium to large mineral particles, bound together by a small amount of organic cement (Fig. 3a, d, e).

#### Biometry

The basic morphometric characters of 75 individuals from the Maromizaha Protected Area in Madagascar were measured and the results are given in Table 1. The analysis of the variation coefficients shows that the studied population of *L. callistoma* is comparatively homogeneous and almost all measured characters are weakly to moderately variable (CV less than 10%). The only exception is the length of the teeth, which varies in a wider range (CV = 12.12%). The parameters with minimal variability are depth of apertural tube (3.37%), depth of shell (3.59%), diameter of shell (4.15%) and diameter at the base of apertural tube (5.32%). The large and small axes of the internal opening are moderate variables (9.20% and 9.13%, respectively). These conclusions are also confirmed by the box plots, showing the variability of the shell’s characters in *L. callistoma* (Fig. 4).

Analysis of the size frequency distribution of the main characters indicates that *L. callistoma* is size-monomorphic species. The presence of comparatively well-expressed main-size class for the basic characters and the lack of the subsidiary peaks (bell-shaped curves) indicate a normal distribution (Fig. 5a, b, c). For example, 93% of all the individuals have a shell diameter of 166-190 µm and only 7% are smaller than 165 µm (Fig. 5a). Almost all measured individuals range in close limits, regarding the shell’s depth and diameter at the base of apertural tube (Fig. 5b, c). 98% of them have a diameter at the base of apertural tube between 54-64 µm, and in more than three quarters of the individuals (82%), shell depth varies between 122-134 µm. The average values (Mean ± SD) for these three shell characters are respectively 175.8 ± 7.30 (n=75), 128.1 ± 4.60 (n=43) and 59.8 ± 3.19 (n=75). These arithmetical means correlate with the main-size classes of the characters, and testify for monomorphism of the species.

## Discussion

Until present, data on the ultramorphology and biometry of *L. callistoma* are almost entirely lacking. In the period from its description to the present time, this species was found only in four countries - Ivory Coast, Papua New Guinea, Philippines and Indonesia (Bonnet 1974, Bonnet 1976, Bonnet 1980a, Bonnet 1980b, Bonnet 1992). Data on the species sizes are given only in its original description. On the basis of measurements of 18 specimens the author gives the next sizes: average shell diameter (Mean ± SD = 175 µm ± 8.1), shell depth (2/3 of the diameter) and apertural invagination (1/3-2/3 of the depth). The morphological data and illustrations of the species are also given only in its original description. In other three publications, where *L. callistoma* was recorded from Papua New Guinea, Philippines and Indonesia data on the species dimensions and illustrations are not given. However in these papers the author published information on the ecology and biogeography of the species and indicated that it is characteristic inhabitant of the humus of acid forest soils. In the biogeographical aspect, the species has been referred to the association of species of the “Gondwanan-tropical” group of soil testate amoebae. In some other publications (Bonnet 1981a, Bonnet 1981b, Bonnet 1984) discusses mainly the biogeographical and palaeogeographic significance of soil testate amoebae. He also included in these discussions information and illustrations about *L. callistoma* based on his previous studies. In these publications the author traces the line *Cyclopyxis-Lamtopyxis-Distomatopyxis* in an evolutionary aspect, emphasizing the trend of flattening on the teeth and reducing of pseudostome. He separated species of the genus *Lamtopyxis* on the basis of the number of teeth and their flatness, as well as of the shape of the internal apertural opening. In almost all papers the author indicated that *L. callistoma* is usually with four flattened teeth and oval internal apertural opening. From the foregoing it is seen that data on the morphology and biometry of species are quite scarce, and in fact the illustrations of the species are based mainly on the drawings, except LM photos of ventral surface of *L. callistoma* given by Bonnet 1979a, Bonnet 1981a.

Biometrical characteristic of the species, based on relatively rich material, was made. In addition to the diameter of the shell, six other shell characters were described biometrically for the first time. Our data for the shell diameter (Mean ± SD) fully corresponds to those given by Bonnet (1974) in the original description of the species (175.8 ± 7.3 (n=75) and 175 ± 8.1 (n=18), respectively). Furthermore, it should be noted that three of the characters measured show high variability (length of teeth, large and small axis of the internal opening). The variability of the last two characters is the reason for the great variability in the shape of the internal opening – from a broad oval to nearly circular.

Surprisingly, our study showed that the population of *L. callistoma* from Madagascar is comprised of both, specimens with four teeth and specimens with three teeth, in ratio of about 60% to 40%. This largely contradicts to the conclusion of Bonnet, who in most of his works emphasized that this species has four teeth. However, even in the original species description the author pointed at that, though rarely, specimens with five teeth have also occurred. Moreover, in his publication for the soil testate amoebae of Papua New Guinea, Bonnet (1980a) stated that in the populations of *L. callistoma* and *L. cassagnaui* specimens with two teeth can be observed very rarely. From the above facts it is seen that the number of teeth cannot be the most reliable taxonomic character for the species separation in the genus *Lamtopyxis*. Moreover, the individuals with three teeth of the external opening are the most typical and are found in all five species of this genus. Maybe for *L. callistoma* specimens with 4 teeth are more frequent, but specimens with three, five and rarely two teeth can also be observed. So, the separation of the species must be made on the base of a complex assessment of many characters: diameter and depth of shell; number, structure and shape of the teeth; sizes and shape of internal aperture. In this regard, from the five known species of the genus *Lamtopyxis* the most distinctive is *L. sarocchii*, with its largest dimensions (Dm = 278-290 µm) and the structure of the apertural opening which is closer to the genus *Cyclopyxis* than to genus *Lamtopyxis* (Coûteaux and Chardez 1981). *L. travei* has similar sizes as *L. callistoma* (Dm = 168-171 µm), but differs from the last by its characteristic elongated shape of the internal opening and by less-developed teeth of the external opening (Bonnet 1977b). The other two species – *L. cassagnaui* and *L. trifoliata* are characterized by three highly developed, flattened and sometimes nearly touching teeth on the external opening and differ from *L. callistoma* by significantly smaller dimensions – an average Dm of about 100 µm (Bonnet 1977b, Bonnet 1979a). *L. trifoliata* has a similar elliptical shape of the internal opening as *L. callistoma*, while *L. cassagnaui* differs significantly from all other species of the genus by its triangular shape of the internal opening.

Another interesting feature observed in our SEM studies of *L. callistoma* is that the thick layer of organic cement, of which the apertural shell surface is composed, extends over the teeth which are made mainly by organic cement. This finding differs from that in the original description of the species, where it is stated that the opening in the base of apertural invagination is surrounded by four large, thickened and rounded “siliceous” teeth.

In respect to the biogeography of *L. callistoma* it is evident that this is a very rare species of the Gondwanan-tropical group of testate amoebae, which has been found only in soils of tropical forests from countries situated south of the Tropic of Cancer. Despite numerous studies on soil testate amoebae carried out in the Northern Hemisphere, it has never been reported from Europe, North America and Asia. Taking into account the restricted geographical distribution, the large sizes and characteristic apertural morphology of *L. callistoma* we assume that this species, like some bryophilic ‘Nebelas’ with circumaustral distribution (e.g. *Apodera vas*, *Alocodera cockayni*, *Certesella certesi*, *Certesella martiali*, etc.), can be used as an example that in free-living microbial eukaryotes ‘not everything is everywhere’.

## Supplementary Material

Supplementary material 1 Lamtopyxis callistoma Data type: Biometry_Stattistica
File: oo_31598.STA Milcho Todorov

XML Treatment for Lamtopyxis callistoma

## Figures and Tables

**Figure 1a.**
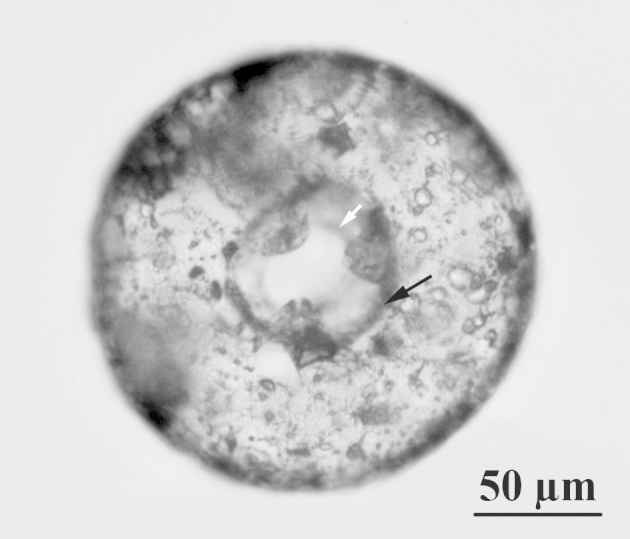
Ventral view of specimen with three teeth, showing oval internal opening at the bottom of the apertural tube (white arrow) and dark coloured organic frame at the base of the tube (black arrow)

**Figure 1b.**
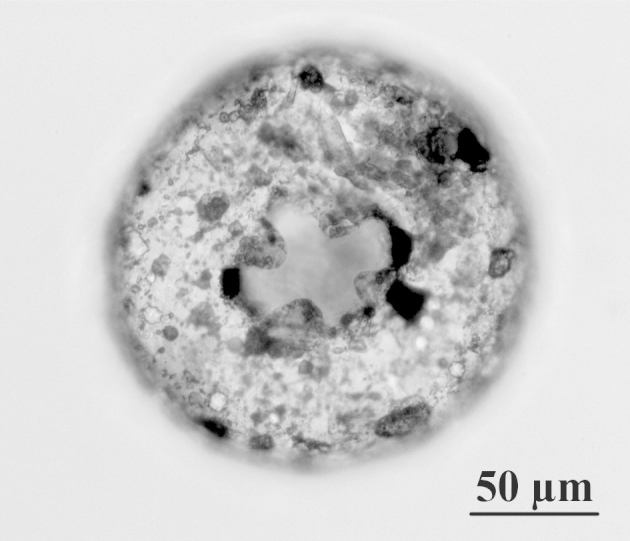
Ventral view of specimen with four teeth

**Figure 1c.**
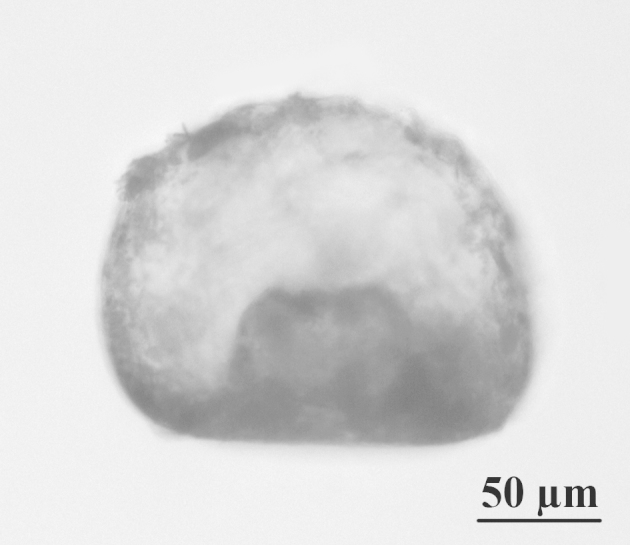
Lateral view of the specimen from Fig. 1a, showing invagination of the apertural tube

**Figure 1d.**
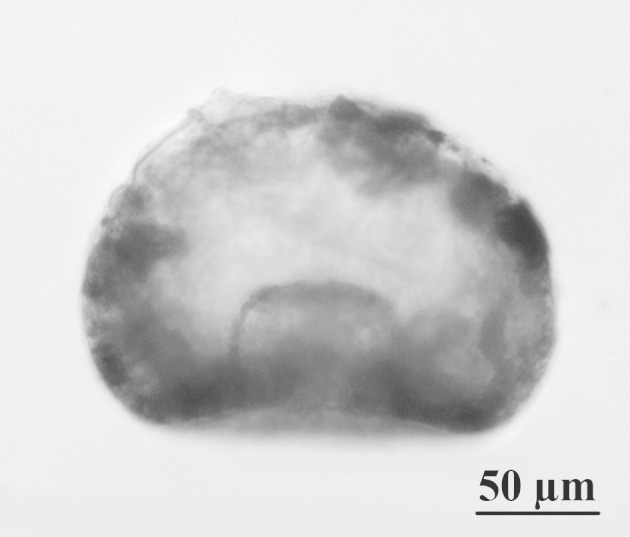
Lateral view of the specimen from Fig. 1b, showing invagination of the apertural tube

**Figure 2a.**
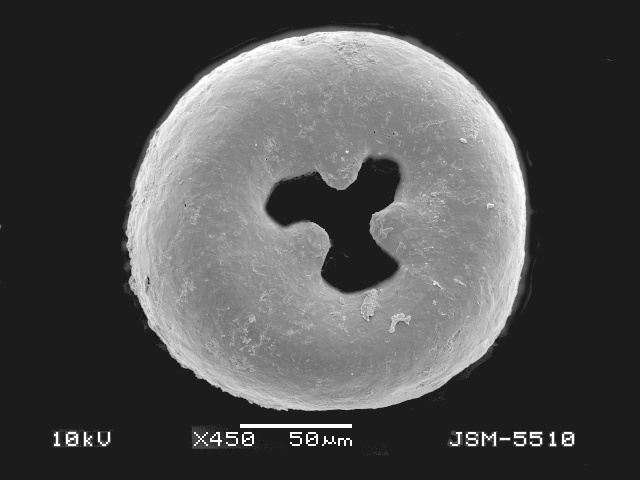
Ventral view of specimen with three teeth

**Figure 2b.**
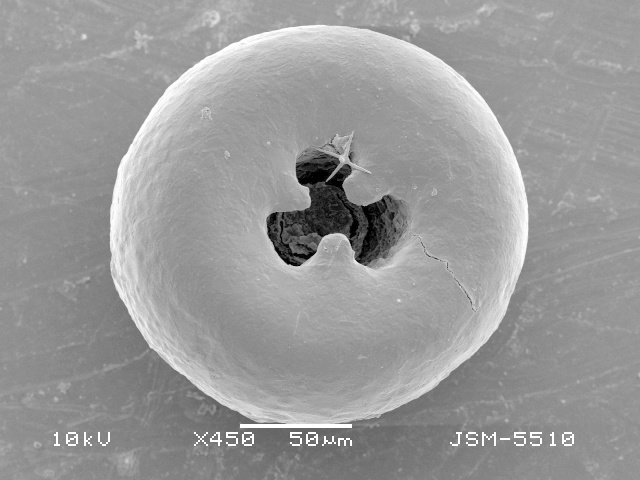
Ventral view of other specimen with three teeth

**Figure 2c.**
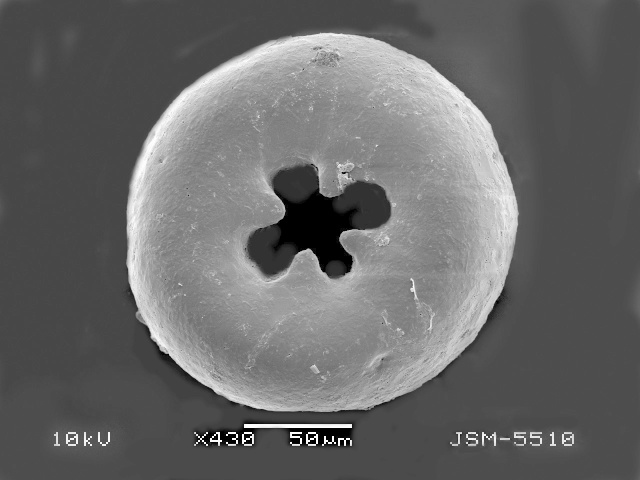
Ventral view of specimen with four teeth

**Figure 2d.**
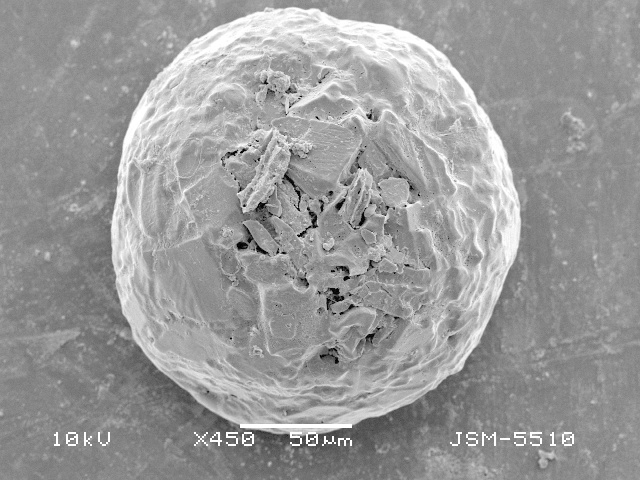
Dorsal view, showing rough dorsal surface of the shell

**Figure 2e.**
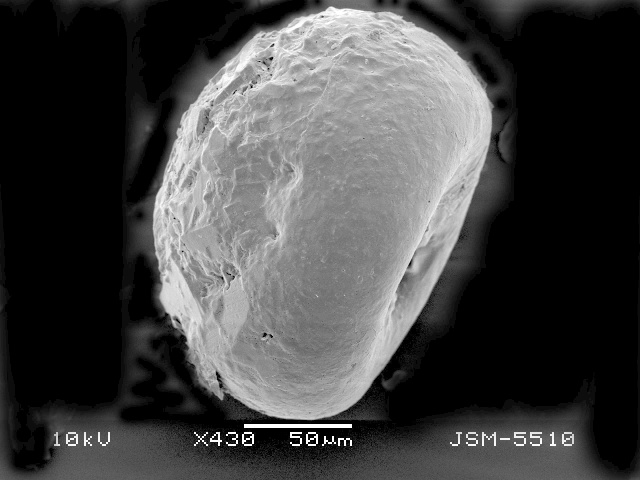
Lateral view, showing rough dorsal and smooth apertural surface of the shell

**Figure 2f.**
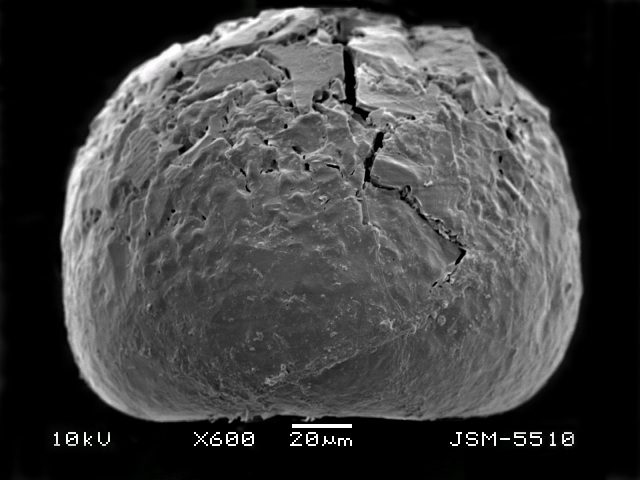
Lateral view, showing rough dorsal and smooth apertural surface of the shell

**Figure 3a.**
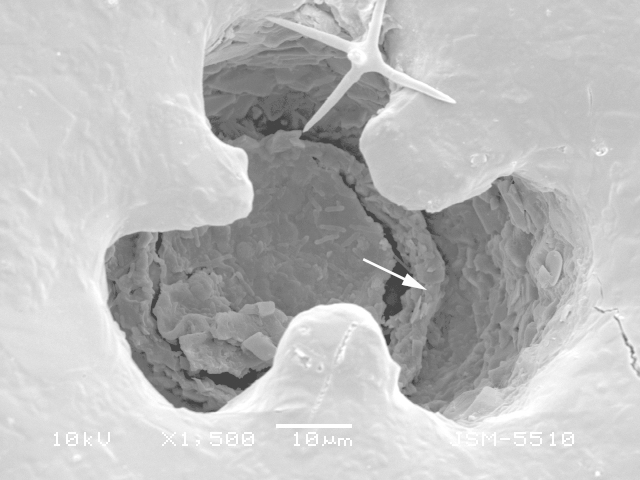
View of aperture to show the smooth teeth and internal opening with collar (arrowed) on the bottom of the apertural tube

**Figure 3b.**
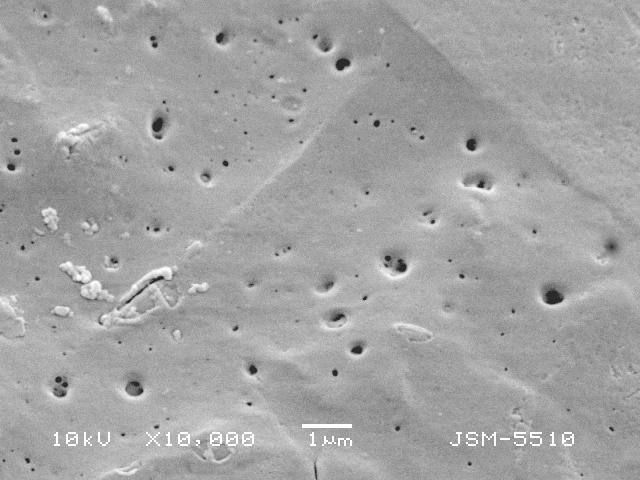
Details of organic cement with single and different sized pores

**Figure 3c.**
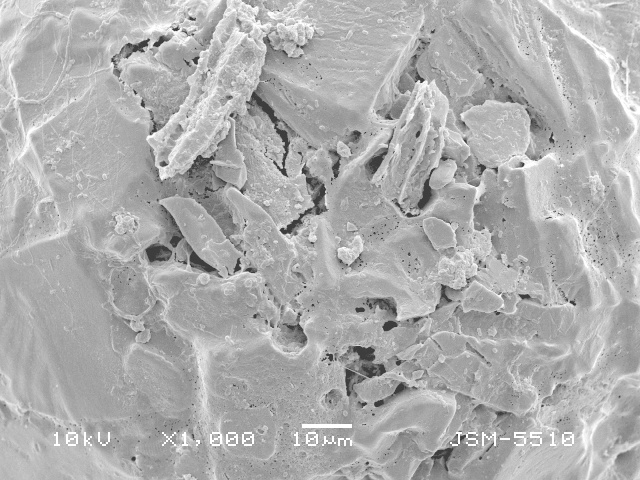
Dorsal view, showing rough dorsal surface of the shell

**Figure 3d.**
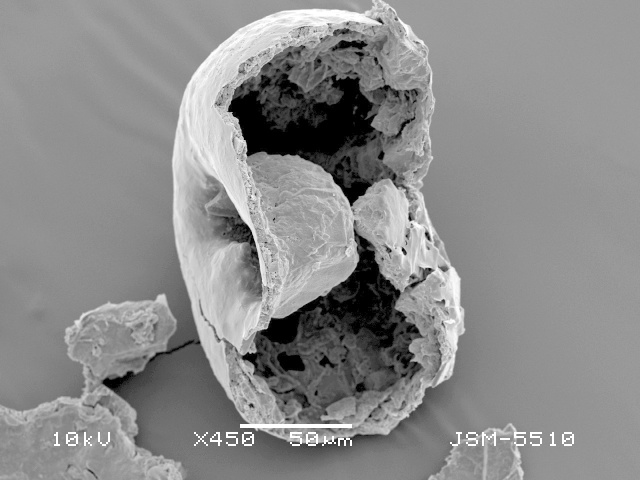
Lateral view of fractured shell, showing deep apertural invagination and its rough surface

**Figure 3e.**
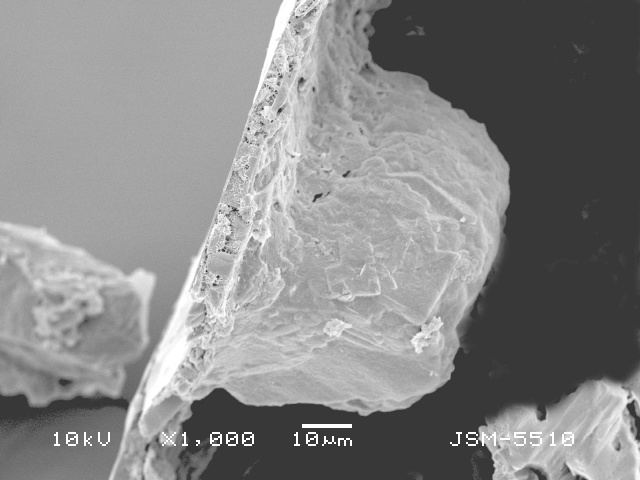
Lateral view of farctured shell, showing deep apertural invagination and its rough surface

**Figure 3f.**
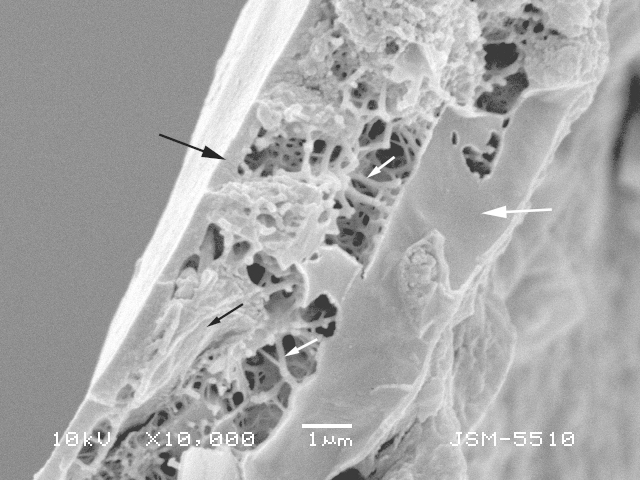
Fractured edge of the shell illustrating the shell structure with smooth outer thin layer of organic cement (large black arrow), intermediate layer of porous and fibrous organic cement (small white arrows) with incorporated mineral particles (small black arrow) and inner thick layer of organic cement (large white arrow)

**Figure 4a.**
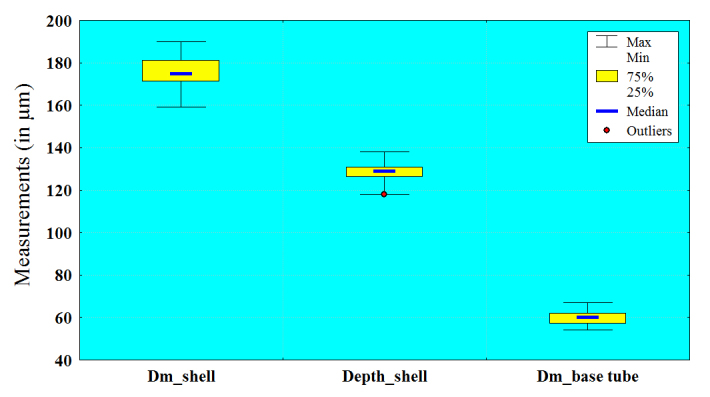
Diameter of shell, depth of shell, diameter at the base of apertural tube

**Figure 4b.**
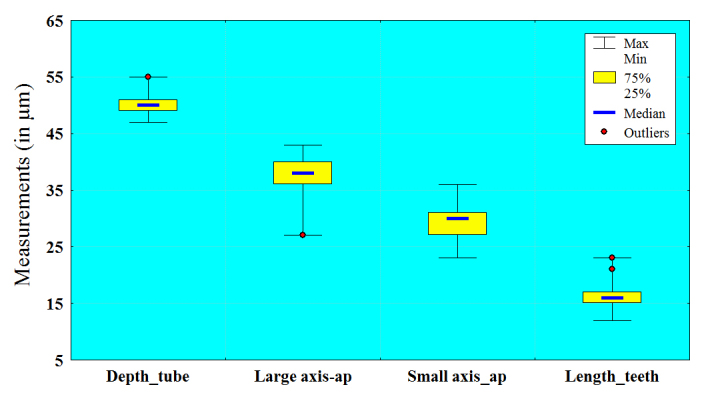
Depth of apertural tube, large axis of internal opening, small axis of internal opening, length of teeth

**Figure 5a.**
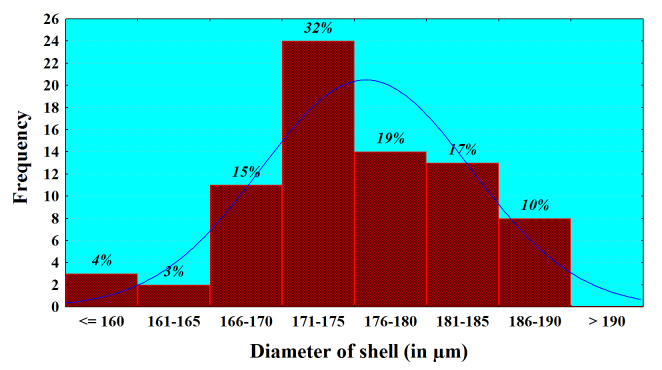


**Figure 5b.**
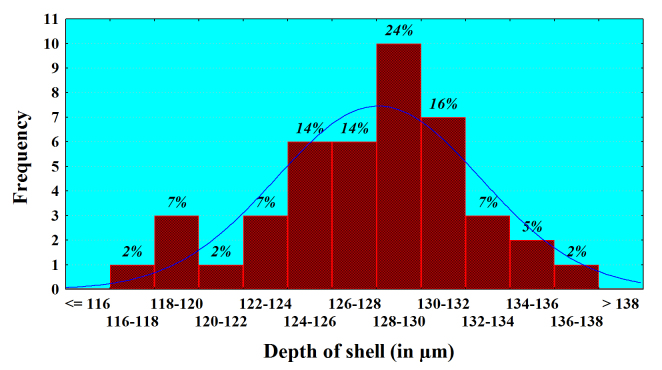


**Figure 5c.**
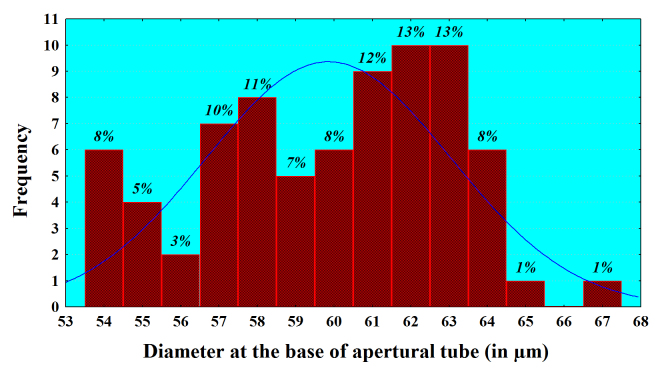


**Table 1.** Biometrical characterization of *Lamtopyxis callistoma*: M – median; SD – standard deviation; SE – standard error of the mean; CV – coefficient of variation in %; Min – minimum; Max – maximum; n – number of individuals examined (measurements in μm).

**Characters**	**Mean**	**M**	**SD**	**SE**	**CV**	**Min**	**Max**	**n**
**Diameter of shell**	175.8	175.0	7.30	0.84	4.15	159	190	75
**Depth of shell**	128.1	129.0	4.60	0.70	3.59	118	138	43
**Diameter at the base of apertural tube**	59.8	60.0	3.19	0.37	5.32	54	67	75
**Depth of apertural tube**	50.0	50.0	1.69	0.26	3.37	47	55	43
**Large axis of internal opening**	37.1	38.0	3.41	0.41	9.20	27	43	69
**Small axis of internal opening**	29.2	30.0	2.67	0.32	9.13	23	36	69
**Length of teeth**	16.2	16.0	1.97	0.23	12.12	12	23	75
